# Optimization of Extraction of Novel Pectinase Enzyme Discovered in Red Pitaya (*Hylocereus polyrhizus*) Peel

**DOI:** 10.3390/molecules181114366

**Published:** 2013-11-20

**Authors:** Nor Khanani Zohdi, Mehrnoush Amid

**Affiliations:** Department of Food Technology, Faculty of Food Science and Technology, Universiti Putra Malaysia, 43400 UPM Serdang, Selangor, Malaysia; E-Mail: norkhanani@gmail.com

**Keywords:** fruit enzyme, specific activity, temperature stability, storage stability, surfactant agent stability

## Abstract

Plant peels could be a potential source of novel pectinases for use in various industrial applications due to their broad substrate specificity with high stability under extreme conditions. Therefore, the extraction conditions of a novel pectinase enzyme from pitaya peel was optimized in this study. The effect of extraction variables, namely buffer to sample ratio (2:1 to 8:1, X_1_), extraction temperature (−15 to +25 °C, X_2_) and buffer pH (4.0 to 12.0, X_3_) on specific activity, temperature stability, storage stability and surfactant agent stability of pectinase from pitaya peel was investigated. The study demonstrated that the optimum conditions for the extraction of pectinase from pitaya sources could improve the enzymatic characteristics of the enzyme and protect its activity and stability during the extraction procedure. The optimum extraction conditions cause the pectinase to achieve high specific activity (15.31 U/mg), temperature stability (78%), storage stability (88%) and surfactant agent stability (83%). The most desirable conditions to achieve the highest activity and stability of pectinase enzyme from pitaya peel were the use of 5:1 buffer to sample ratio at 5 °C and pH 8.0.

## 1. Introduction

Pectic enzymes or pectinases alone account for about one quarter of the World’s food enzyme production and are among the most important industrial enzymes [1]. They are of great significance, with a wide range of applications in the fruit and beverage and textile processing industries, in the treatment of pectin wastewaters, degumming of plant fibers, pulp and papermaking, and for coffee and tea fermentation [2]. Most of the commercial production of pectinases is limited to some species of bacteria, yeast and fungi [3]. Due to the extensive applications of the enzyme in various industries there is a need to find novel sources of the enzymes due to their current limited production. Furthermore, utilization of agro-industrial residues for enzyme production minimizes pollution and allows the production of high value-added products using an economical process [4]. Pitaya or dragon fruit (*Hylocereus polyrhizus*) is among the important commercial tropical fruits in the World [5,6]. Pitaya Peel accounts for around 33% of whole fruit weight [7,8], but it is not currently used in any commercial process and thus ends up as waste and a source of pollution. There are different types of enzymes in pitaya peel such as pectinase and thus it could be used as a rich and cost effective source for the commercial production of natural and valuable kinds of the enzymes. Alteration or destruction to the natural morphology of the tertiary structure of protein during the extraction process causes a decrease in the activity and stability of enzymes [9], therefore enzyme extractions should be performed under optimized conditions in order to achieve high enzyme activity and stability. The main objective of the present study was to investigate the effect of extraction variables on the enzymatic properties of pectinase from pitaya peel. Subsequently, the optimization of the extraction condition process resulted in achieving the maximum specific activity, temperature stability and pH stability. The pectinase extraction variables included buffer to sample (B/S) ratio (2:1 to 8:1, w/w) extraction temperature (−15 to 25 °C) and buffer pH (4.0 to 12.0). It should be noted that the optimisation of extraction conditions of pectinase from pitaya peel with the most desirable and appropriate enzymatic properties has not been done to date.

## 2. Results and Discussion

### 2.1. Fitting the Final Reduced Models

In the study, multiple regression analysis was carried out using a response surface analyser to establish a relationship between three enzyme extraction variables and the enzymatic properties of pectinase extracted from pitaya peel. It should be noted that in the final reduced model just significant (*p* < 0.05) terms were kept. The non-significant terms were kept when their quadratic or interaction effect showed a significant (*p* < 0.05) effect on the response variables. The Equations (1–4) show the main quadratic and interaction effects of the factors affecting the response variables. The estimated regression coefficient of independent variables, as well as *R*^2^ and lack of fit are shown in Table 1. The results indicate that the *R*^2^ values for pectinase specific activity, thermal stability; storage stability and surfactant agent stability were 0.984, 0.978, 0.889 and 0.980, respectively. Thus, based on the results all *R*^2^ values for all response variables were more than 80%, therefore the response surface models were suitably and accurately used to predict the properties of extracted enzyme as a function of the extraction variables. The lack of fit, indicating the fitness of models, showed no significant *p*-value (*p* > 0.05) in terms of response variables studied at 95% confidence level, thus confirming the sufficient fitness of the regression model with the experimental values. The significant (*p* < 0.05) effect of each term was determined using the F-ratio and *p*-value as presented in Table 2. According to the results, the main effect of B/S ratio and temperature showed the most and least significant effects on the response variables, respectively. In addition, the interaction effect of B/S ratio and pH of buffer had the most significant effect among the other interaction effects. The following response surface models (Equations (1–4)) were fitted to each of the response variables (Y), and three independent variables (X_1_, X_2_ and X_3_):

Y1 = 15.14 + 3.5X_1_ + 5.50X_2_ + 2.52X_1_^2^ + 1.88 X_2_^2^ + 1.92X_3_^2^ + 5.36X_1_X_3_ + 2.50X_2_X_3_ (1)

Y_2_ = 77.01 + 13.33X_2_ − 11.72X_3_ + 18.11X_2_^2^ + 9.40 X_3_^2^ + 11.73X_1_X_3_ (2)

Y_3_ = 81.74 + 15.20X_2_ + 11.74X_3_ + 25.04X_2_^2^ + 13.38X_3_^2^ + 27.24X_2_X_3_ (3)

Y_4_ = 79.82 + 24.70X_2_ + 14.31X_3_ + 24.04X_2_^2^ + 15.57X_3_^2^ + 26.95X_2_X_3_ (4)

**Table 1:** Regression coefficients, *R^2^*, *p*-value of lack of fit for the Polynomial Response Surface.

Regression coefficient	Specific activity (Y_1_)	Temperature stability (Y_2_)	Storage stability (Y_3_)	Surfactant agent stability (Y_4_)
*b_0_*	15.14	77.01	81.74	79.82
*b_1_*	3.50	11.62	24.58	12.96
*b_2_*	5.50	13.33	15.20	24.70
*b_3_*	0.53	−11.72	11.74	14.31
*b_1_^2^*	2.52	4.15	4.38	8.65
*b_2_^2^*	1.88	18.11	25.04	24.04
*b_3_^2^*	1.92	9.40	13.38	15.57
*b_1_b_2_*	0.51	12.30	4.90	5.49
*b_1_b_3_*	5.36	11.73	13.02	21.04
*b_2_b_3_*	2.50	17.73	27.24	26.95
*R^2^*	0.984	0.978	0.889	0.980
*p*-value	0.001 *	0.001 *	0.004 *	0.002 *
Lack of fit (*p-*value)	105.77	195.10	102.99	111.87

^1^, B/S ratio; ^2^, Temperature; ^3^ pH of buffer; * Significant (*p* < 0.05); *b*_i_, *b*_ii_ and *b*_ij_: the estimated regression coefficient for the main linear quadratic and interaction effects, respectively.

### 2.2. Specific Activity of Pectinase

As shown in Table 2, the main effect of all independent variables (*i.e.*, B/S ratio, temperature and pH of buffer) as well as all quadratic effects of variables indicated a significant (*p* < 0.05) effect on the specific activity of the enzyme. In addition, the interaction effect of B/S ratio with buffer pH and the interaction effect of temperature with buffer pH also had a significant (*p* < 0.05) effect on the specific activity of the pectinase enzyme (Table 2).

**Table 2:** F-ratio and *p*-value for each Independent Variable Effect in the Polynomial Response Surface Models.

Variables		Main effects			Quadratic effects			Interaction effects		
		X_1_	X_2_	X_3_	X_1_^2^	X_2_^2^	X_3_^2^	X_1_X_2_	X_1_X_3_	X_2_X_3_
Specific activity(Y_1_, U/mg)	*p*-value	0.000 *	0.003 *	0.187	0.000 *	0.024 *	0.001 *	0.057	0.003 *	0.016 *
	F-ratio	110.88	45.42	2.52	109.66	12.53	64.48	4.84	42.12	16.16
Temperature stability (Y_2_, %)	*p*-value	0.066	0.026 *	0.005 *	0.100	0.002 *	0.006 *	0.055	0.018 *	0.124
	F-ratio	3.24	9.79	22.94	4.04	33.64	20.88	7.18	11.83	2.56
Storage stability (Y_3_, %)	*p*-value	0.061	0.017 *	0.039 *	0.191	0.013 *	0.009*	0.454	0.103	0.047 *
	F-ratio	6.70	15.21	9.06	2.46	17.80	23.04	0.67	4.44	8.00
Surfactant agent stability (Y_4_, %)	*p*-value	0.051	0.027 *	0.002 *	0.126	0.030 *	0.008 *	0.064	0.050	0.004 *
	F-ratio	5.18	11.76	54.02	3.68	10.82	23.42	1.21	8.94	35.88

X_1_, X_2_ and X_3_: The main effect of B/S ratio, temperature and pH of buffer, respectively. X_1_^2^, X_2_^2^ and X_3_^2^: The quadratic effect of B/S ratio, temperature and pH of buffer, respectively. X_1_X_2_: The interaction effect of B/S ratio and temperature. X_1_X_3_: The interaction effect of B/S ratio and pH of buffer and X_2_X_3_: The interaction effect of temperature and pH of buffer. * Significant at (*p* < 0.05).

Based on the results (Table 2), the main effect of temperature shows that the most (*p* < 0.05) significant effect is based on the F-ratio (110.88) of this independent variable. It confirms that the enzyme is heat sensitive and the activity is decreased at high or low temperature due to denaturation of the tertiary structure of pectinase.

**Figure 1:**
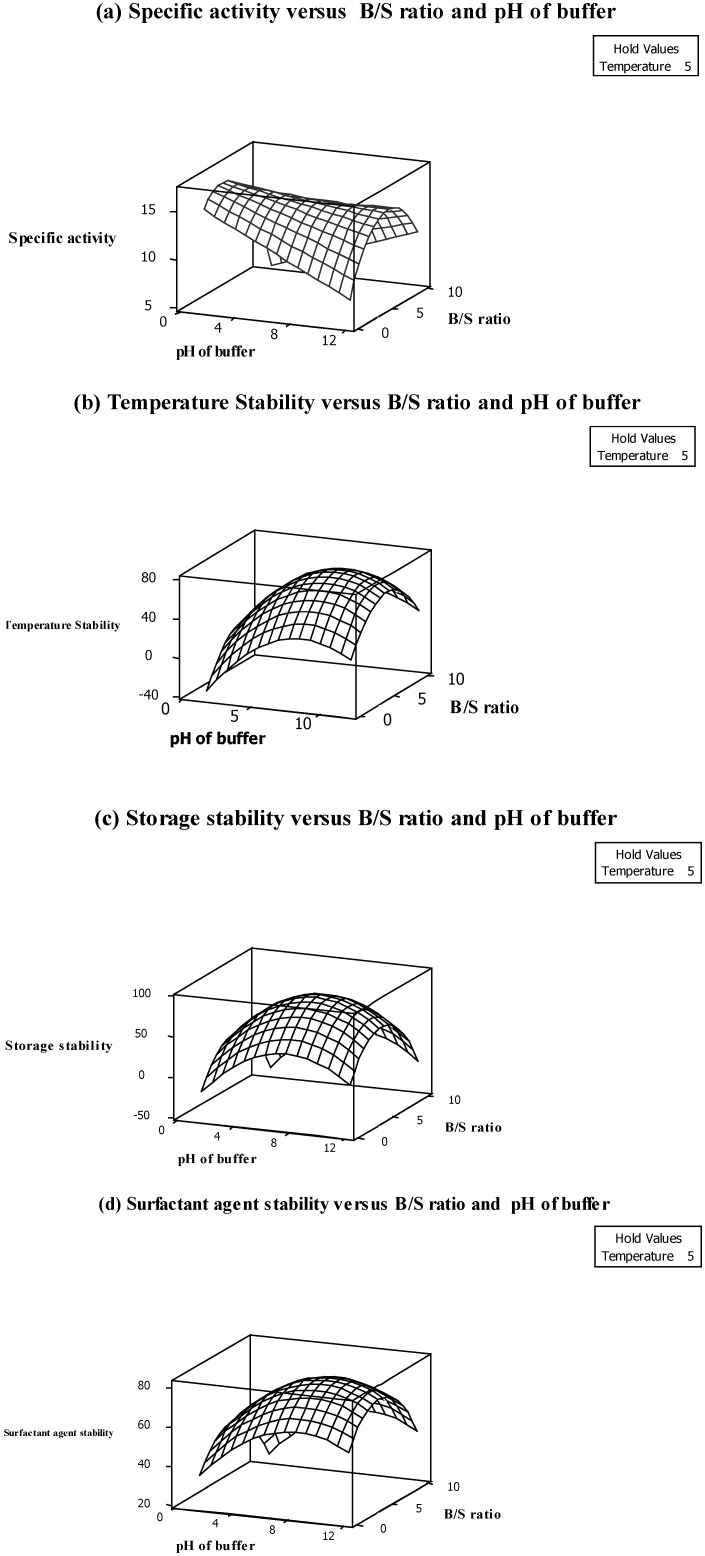
Response surface plots showing the interaction effects of (**a**) specific activity, (**b**) Thermal stability, (**c**) Storage stability (**d**) Surfactant agent stability.

Figure 1a shows that pectinase specific activity is increased by increasing the B/S ratio from 2:1 to 5:1. In fact, increasing the B/S ratio increases the pectinase activity because of greater binding capacity buffering of the active site of pectinase (Figure 1a). It should be noted that the enzyme activity is decreased in lower amounts of buffer because of the difficulty in homogenizing the sample with buffer and also the decreasing enzyme solubilisation in crude extract [10]. The pectinase also showed the highest specific activity at pH 5.0. As shown in Figure 1a, the enzyme activity is significantly (*p* < 0.05) decreased at pH 2.0 and 8.0 due to the unfolding and decomposition of the enzyme structure in low acidic and alkaline pH. In fact, enzymes have ionic groups in their active site which must be in a stable form. Variation in pH of the medium results in changes in the ionic form of the active site which affects the reaction rate and decreases enzyme activity [11]. As shown in Table 3, the highest specific activity of pectinase (15.31 U/mg) was obtained when the pectinase was extracted at 5 °C temperature, B/S ratio 3:1 and pH of buffer 5.0.

### 2.3. Temperature Stability of Pectinase

Based on the results (Table 2), the main effect of all independent variables excluding temperature significantly (*p* < 0.05) influenced the extraction of pectinase from pitaya peel. In addition, the quadratic and interaction effects of temperature on other variables also did not show any significance on pectinase temperature stability. In fact, the enzyme retained around 78% of stability at different extraction temperatures (Table 3). It can be therefore be inferred that the enzyme is stable at different extraction temperatures ranging from freezing temperature to room temperature. It should be noted that temperature stability of the pectinase is one of the good characteristics of the enzyme. The advantages of thermostable pectinases, especially in industrial processes, include decreased risk of contaminants and also cost of external cooling, increased substrate solubility and a lower viscosity and allowance for accelerated mixing [12]. Additionally, the temperature stability was positively proportional to the main effect of B/S ratio which was negatively proportional to buffer pH (Table 2). This means that an increase in B/S ratio from 2:1 to 5:1 increases the temperature stability while increasing the pH to pH 8.0 decreases the pectinase thermostability (Figure 1b). In addition, the interaction effect of B/S ratio and buffer pH had a significant (*p* < 0.05) effect among the other interaction effects (Table 2). The 3D surface plot was plotted to visualize the significant (*p* < 0.05) interaction effect of enzymatic extraction variables on temperature stability (Figure 1b). As shown in Figure 1b the temperature stability of the enzyme at different extraction temperatures was increased by increasing the B/S ratio. In fact, the temperature stability of the enzyme was increased with the simultaneous increase in B/S ratio and increase of buffer pH up to a certain level. Thus, the higher level of the buffer pH above the optimum points which are found during the extraction procedure decreased the temperature stability of the pectinase. However, pectinase from pitaya peel showed that it is not stable at pH 8.0 (alkaline pH) which decreases the enzyme stability toward temperature (Figure 1b). The other interaction effects which included the variable of temperature did not show any significant effect, which confirmed the enzyme’s temperature stability (Table 2).

**Table 3:** The experimental data obtained for the response variables.

Treatment	Temperature (X1)	Buffer to sample ratio (X2)	pH of buffer (X3)	Specific activity (U/mg)	Temperature stability (%)	Storage stability (%)	Surfactant agent stability (%)
1	−15	8:1	4.0	4.4	38.2	42.1	37.3
2	−15	2:1	12.0	6.2	26.3	34.2	28.1
3	25	8:1	12.0	10.4	62.1	73.2	66.1
4	25	2:1	4.0	7.3	51.7	56.8	47.2
5 ^c^	5	5:1	8.0	15.3	78.0	88.1	82.5
6 ^c^	5	5:1	8.0	14.2	77.8	87.5	83.0
7 ^c^	5	5:1	8.0	15.3	78.0	88.0	83.0
8	25	8:1	4.0	9.2	58.3	62.1	54.1
9 ^c^	5	5:1	8.0	15.1	77.6	88.0	82.2
10	−15	2:1	4.0	3.1	17.2	23.4	26.1
11	−15	8:1	12.0	5.8	45.1	47.2	41.4
12	25	2:1	12.0	8.4	34.1	41.5	33.2
13 ^c^	5	5:1	8.0	15.2	78.0	87.9	83.0
14	5	9:1	8.0	12.1	69.2	70.3	71.2
15	−27	5:1	8.0	1.1	10.2	11.1	14.3
16	37	5:1	8.0	2.3	14.0	21.4	17.2
17	5	1:1	8.0	12.3	67.1	61.1	62.2
18	5	5:1	1.4	8.2	53.1	50.1	56.5
19	5	5:1	11.2	11.2	66.4	53.2	49.2
20 ^c^	5	5:1	8.0	15.3	78.0	88.0	82.4

^c^, center point.

### 2.4. Storage Stability of Pectinase

Achieving high storage stability is one of the most important parameters which should be considered in the extraction procedure. According to the results shown in Table 2, the main effect of pH and interaction effect of B/S ratio with buffer pH are positively related to the storage stability of pectinase from pitaya peel. On the contrary, the main, quadratic as well as interaction effects of temperature on other independent variables do not show any significant effect on the storage stability of pectinase from pitaya peel. This is a desirable characteristic for the enzyme which shows that the enzyme is stable at different temperatures. The 3D surface plots were plotted to visualize the significant interaction effecte of extraction variables. Based on Figure 1c, storage stability was increased by simultaneously increasing the B/S ratio from 2:1 to 5:1 and increasing the buffer pH to pH 5.0. In fact, buffer concentration alters the enzyme microenvironment through increased compactness of the protein structure [13]. In addition, high storage stability at pH 8.0 indicates that the active site of the enzyme is stable at alkaline pH and could interact properly with substrates after storage time. The tertiary structure of the enzyme is unfolded and denatured at extremely low pH and also at strongly alkaline pH values (*i.e.*, pH 4.0 and 12.0). A similar observation was reported by Celestino *et al.* [14], who purified and characterized a novel pectinase from *Acrophialophora nainiana*. They also noted that the enzyme stability significantly decreased at acidic pH and pH 9.0. As clearly shown in Table 3, the highest storage stability (88%) of pectinase from pitaya peel after one week storage at 5 °C was obtained at pH 8.0 using a five times the concentration B/S ratio.

### 2.5. Surfactant Agent Stability

Surfactant agent stability of the enzyme is one of the important parameters enabling enzymes to be used in different types of industries, especially the detergent industry [15]. Thus, achieving the highest surfactant agent stability of the enzyme is one of the main goals for optimization of any pectinase extraction procedure. Most of the surfactants which interact with proteins cause distinct electrostatic and hydrophobic regions and alter the secondary or tertiary structure of enzymes [16]. Therefore, the stability of some enzymes is markedly decreased in the presence of surfactant agents, while extracted pectinase from pitaya peel under optimum condition retained 83% of its stability, which is a significant achievement in the study. According to the results (Table 1), the final reduced model fitted the surfactant agent stability and showed a relatively high *R^2^* with no indication of significant (*p* > 0.05) lack of fit. This indicates a satisfactory fitness of the surfactant agent stability model as a function of enzymatic extraction variables (Table 1). As shown in Table 2, the main effect of B/S ratio had a negative effect on surfactant agent stability of pectinase from pitaya peel based on the F-ratio (54.02), while the interaction effect of B/S ratio with buffer pH had a positive effect on the response variable. It confirmed that B/S ratio had both positive and negative effects on the surfactant agent stability of the enzyme. As shown in Figure 1d increasing the B/S ratio up to a certain point increases the stability but, further increases of the B/S ratio decreases the stability of the enzyme. The stability of enzyme in the presence of surfactant at acidic pH was decreased, which could be due to reduction in the hydrophobic interaction which plays an important role in holding together the tertiary protein structure [4].

### 2.6. Optimization Procedures

In the study, the most suitable extraction condition of the pectinase is considered the optimum point if the extraction of the enzyme results in the highest enzyme specific activity, temperature stability, surfactant agent stability as well as storage stability. Overall, optimum extraction conditions were obtained by running multiple graphical and numerical optimizations. Multiple graphical optimizations were carried out by drawing the overlaid counter plot to determine the overall optimum region of the pectinase extraction conditions. Therefore, the extraction conditions under the recommended optimum condition resulted in the extraction of the pectinase from pitaya peel with desirable enzymatic properties. For the purpose of the graphical optimization process, it was proposed that the 3D response surface plotting be used (Figure 1a–d), followed by superimposing all 3D plots to determine the optimum conditions [17]. The 3D plots were generated within the experimental range by fixing and varying a centre point and two variables. With the aid of the response optimizer, a numerical optimization was performed to determine the exact optimal levels of individual and simultaneous multiple response optimizations in order to achieve the desired response goals. Furthermore, in order to determine the adequacy of the response surface equations, a comparison was made between the experimental data and predicted values from the reduced response regression. The results indicated that the extraction using B/S ratio at a concentration of 5:1, at pH 8.0 and at 5 °C for 4 min provided the overall optimum region in terms of all pectinase properties (Table 3).

### 2.7. Model Validation

**Figure 2:**
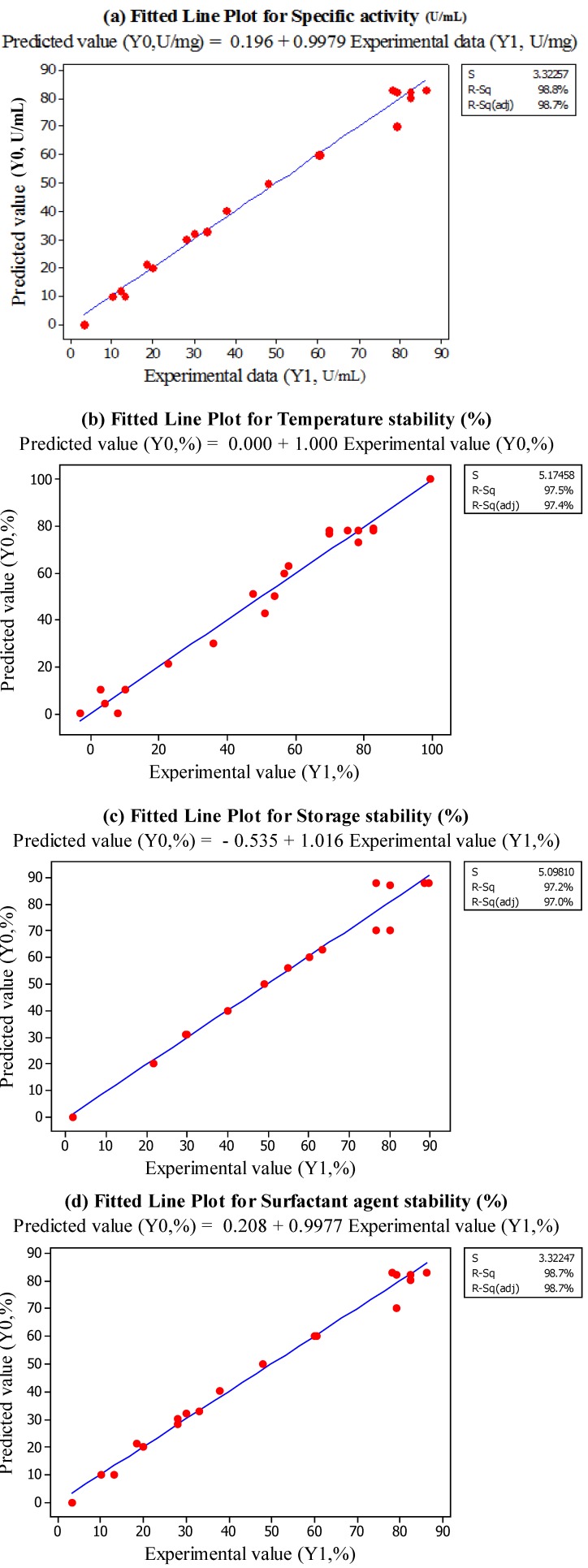
Fitted Line Plots for Predicted Value (Y1) and Experimental Data (Y0). (**a**) specific activity, (**b**) temperature stability, (**c**) Storage stability, (**d**) surfactant stability.

Response surface equation adequacy is shown by comparing the experimental value and the predicted data [18], which is performed by generating a fitted-line plot (with experimental values on X-axis and predicted values on Y-axis) for the obtained results, showing its closeness to or deviation from the fitted line. Figure 2a–d show the overall closeness of these variables, thus indicating that the response surface model is adequate for predicting the varied enzymatic properties as functions of the conditions in extraction. Thus, based on the result, the optimum points for B/S ratio, temperature and buffer pH are 1:4, 5 °C and pH 8.0, respectively.

## 3. Experimental

### 3.1. Chemicals and Plant Material

All chemicals and reagent were analytical grade. Bradford Reagent, bovine serum albumin (BSA), polygalacturonic acid, and 3,5-dinitrosalicylic acid (DNS) were obtained from Sigma Chemical Co. (St. Louis, MO, USA). Dibasic sodium phosphate (Na_2_HPO_4_·2H_2_O), monobasic sodium phosphate (NaH_2_PO_4_·H_2_O), sodium acetate, acetic acid, sodium citrate, citric acid, soluble starch, maltose, sodium potassium tartrate (NaKC_4_H_4_O_6_∙4H_2_O) was obtained from Merck (Darmstadt, Germany). Red pitaya fruits (*Hylocereus polyrhizus*) were purchased from Passer Borong (Selangor, Malaysia). Ripened pitaya fruits free of visual defects were selected based on the size uniformity at the same stage of ripening. The fruits were stored in a cold room at 4 °C until used for the extraction procedure.

### 3.2. Extraction of Pectinase from Pitaya Peel

Pitaya fruits were washed with distilled water, peeled with a stainless steel knife and cut into small pieces. Subsequently, the sample was blended (Model 32BL80, Dynamic Corporation of America, New Hartford, CT, USA) with buffer under enzyme extraction conditions, *i.e.*, buffer to sample ratio B/S ratio (2:1 to 8:1), temperature (−15 °C to +25 °C) and pH (4.0–8.0). In the experiments, the concentration and ionic strength of the buffers used the following buffer solutions: 0.1 M glycine–HCl (pH 1.0–3.5), 0.1 M sodium acetate (pH 4.0–5.5); 0.1 M sodium phosphate (pH 6.0–7.5); 0.1 M Tris–HCl (pH 8.0–9.0); 0.1 M glycine-NaOH (pH 9.5–12). The homogenate was filtered through cheesecloth and the filtrate was centrifuged at 8,000 rpm, for 10 min at 4 °C. The extracted enzyme was kept at 4 °C for future studies.

### 3.3. Pectinase Activity Assay

Pectinase activity was measured by determination reduction groups released from polygalacturonic acid as substrate. The reaction mixture contained the enzyme (0.5 mL) and polygalacturonic acid (0.5 mL) which is dissolved in 100 mM acetate buffer at pH 4.5. The mixture was incubated at 37 °C for 30 min in water batch. After incubation, DNS (1 mL) was added to the mixture to stop the reaction and then the sample was boiled for 5 min. The released reducing sugar was determined by spectrophotometry (BioMate^TM^-3, Thermo Scientific, Alpha Numerix, Webster, NY, USA) at 575 nm using galactouronic acid as standard reducing sugar [19]. The results were carried out as a mean of three readings with as estimated error of ±10%.

### 3.4. Protein Concentration Determination

The protein contents of samples were determined using dye binding method as described by Bradford [20] and BSA was used as standard.

### 3.5. Determination of Specific Activity of the Pectinase

Specific activity of pectinase was determined by divided of total pectinase activity to total protein concentration as equation below (Equation (5)) [21]:

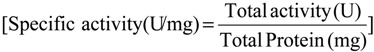
(5)

### 3.6. Determination of Temperature Stability

Thermostability of pectinase from different conditions of extraction was determined by incubation of the enzyme in a 50 mM sodium phosphate buffer (pH 4.5) at different temperatures ranging from 20, 30, 40, 50, 60, 70, 80, 90 and 100 °C for 30 min. After incubation, the residual activity of pectinase was measured by polygalactouronic acid at pH 4.5 at 37 °C [15,22].

### 3.7. Determination of Storage Stability

Pectinase after extraction was stored at cold room temperature (±4 °C) for 10 days. Then, the enzymatic activity of the pectinase after storage was measured as earlier mentioned. The ratio of pectinase activity after storage to the activity of pectinase before storage provided the efficiency of pectinase storage stability as shown in equation below:

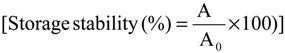
(6)
where A is enzyme activity of pectinase after storage time and A_0_ is initial enzyme activity of pectinase [23].

### 3.8. Determination of Surfactant Agent Stability

The effect of different surfactant agents such as SDS, Tween 80 and Triton X-100 on enzyme stability of pectinase was determined. For this purpose, extracted enzyme (0.5 mL) was incubated in the presence of 5% (w/w) surfactant (0.5 mL) at 37 °C for 30 min. Subsequently, the pectinase activity was determined according to the standard assay conditions [24].

### 3.9. Experimental Design

Response surface methodology (RSM) was used to determine the effect of the enzyme extraction variables, *i.e.*, B/S ratio (2:1 to 8:1), extraction temperature (−15° C to +25 °C) and buffer pH (4.0 to 12.0) on the enzymatic properties of pectinase from pitaya peel (Table 4). Twenty treatments were assigned based on a central composite design with three independent variables at file levels of each variable including six centre points, eighteen factorial points and six star (axial) points. The enzymatic properties of pectinase such as specific activity, thermostability, storage stability and surfactant agent stability were considered as response variables. Experiments were randomized in order to minimize the effects of unexplained variability in the actual responses due to extraneous factors [18].

**Table 4:** Matrix of the Central Composite Design (CCD).

Treatment	Temperature (X_1_)	Buffer to sample ratio (X_2_)	pH of buffer (X_3_)
1	−15	8:1	4.0
2	−15	2:1	12.0
3	25	8:1	12.0
4	25	2:1	4.0
5 ^c^	5	5:1	8.0
6 ^c^	5	5:1	8.0
7 ^c^	5	5:1	8.0
8	25	8:1	4.0
9 ^c^	5	5:1	8.0
10	−15	2:1	4.0
11	−15	8:1	12.0
12	25	2:1	12.0
13 ^c^	5	5:1	8.0
14	5	9:1	8.0
15	−27	5:1	8.0
16	37	5:1	8.0
17	5	1:1	8.0
18	5	5:1	1.4
19	5	5:1	11.2
20 ^c^	5	5:1	8.0

### 3.10. Statistical Analysis

Response surface analysis was performed to determine regression coefficients and statistical significance of the model as well as fit the regression models to the experimental data to achieve an overall optimum region for all response variables studied. The prediction of the optimum pectinase extraction condition was expressed according to the following equation:

*Y = β_0_ + β_1_X_1_ + β_2_X_2_ + β_3_X + β_11_X_1_^2^ + β_22_X_2_^2^ + β_33_X_3_^2^ + β_12_X_1_X_2_ + β_13_X_1_X_3_ + β_23_X_2_X_3_* (7)

where *Y* represents response function, *β_0_* is an intercept, *β*_1_, *β*_2_ and *β*_3_ are the regression coefficients for linear terms, *β*_11_, *β*_22_ and *β*_33_ are quadratic effects, and *β*_12_, *β*_13_ and *β*_23_ are the interaction terms. Accordingly, *X_1_*, *X_2_*, *X_3_* and *X_4_* represent the independent variables. The analysis of variance tables was generated, and the regression coefficients of individual linear, quadratic and interaction terms were considered. It should be noted that the corresponding variables were considered more significant (*p* < 0.05) as the F-ratio became larger and the *p*-value became smaller [18]. The adequacy of the model was measured using analysis of model, lack of fit and determination of coefficient (*R^2^*) [4,17] Kutner *et al.* [25] suggested that a good fit model should possess a minimum *R*^2^ of 0.80.

## 4. Conclusions

The present study indicated that the enzymatic properties of pectinase from pitaya peel were shown to be significantly (*p* < 0.05) affected by the levels of the main extraction variables. The results showed that the activity and stability of the pectinase were significantly influenced by changes of B/S ratio and buffer pH. The least significant (*p* < 0.05) effect of temperature on enzyme activity of the pectinase confirmed that the enzyme is thermostable. Since enzymes are heat sensitive biomolecules, thus, thermostability is one of the most desirable characteristics for the application of an enzyme in various types of industries. The high stability of pectinase in the presence of a surfactant agent is also one of the important characteristics of an enzyme. Based on the results, the main effect of B/S ratio and buffer pH should be considered as important parameters for the extraction of pectinase from fruit source. The study demonstrated that the desirable condition for extraction of pectinase from pitaya peel was using a B/S ratio 5:1, at 5 °C temperature and pH 8.0. The results of the study indicated that the natural and valuable enzyme from pitaya peel with its unique characteristics such as thermostabilty, high stability at alkaline pH and surfactant agent stability can be used as a potential enzyme in different types of industries and biotechnological applications.
